# Determination of Phenolic Acids and Flavonoids in *Taraxacum formosanum* Kitam by Liquid Chromatography-Tandem Mass Spectrometry Coupled with a Post-Column Derivatization Technique

**DOI:** 10.3390/ijms13010260

**Published:** 2011-12-27

**Authors:** Hung-Ju Chen, Baskaran Stephen Inbaraj, Bing-Huei Chen

**Affiliations:** 1 Department of Food Science, Fu Jen University, Taipei 242, Taiwan; E-Mails: a29939245@hotmail.com (H.-J.C.); sinbaraj@yahoo.com (B.S.I.); 2 Graduate Institute of Medicine, Fu Jen University, Taipei 242, Taiwan

**Keywords:** *Taraxacum formosanum* Kitam, phenolic acid, flavonoid, LC-MS-MS

## Abstract

A liquid chromatography-tandem mass spectrometry method (LC-MS/MS) was developed for the determination of phenolic acids and flavonoids in a medicinal Chinese herb *Taraxacum formosanum* Kitam. Initially, both phenolic acids and flavonoids were extracted with 50% ethanol in a water-bath at 60 °C for 3 h and eventually separated into acidic fraction and neutral fraction by using a C_18_ cartridge. A total of 29 compounds were separated within 68 min by employing a Gemini C_18_ column and a gradient solvent system of 0.1% formic acid and acetonitrile at a flow rate of 1.0 mL/min. Based on the retention behavior as well as absorption and mass spectra, 19 phenolic acids and 10 flavonoids were identified and quantified in *T. formosanum*, with the former ranging from 14.1 μg/g to 10,870.4 μg/g, and the latter from 9.9 μg/g to 325.8 μg/g. For further identification of flavonoids, a post-column derivatization method involving shift reagents such as sodium acetate or aluminum chloride was used and the absorption spectral characteristics without or with shift reagents were compared. An internal standard syringic acid was used for quantitation of phenolic acids, whereas (±) naringenin was found suitable for quantitation of flavonoids. The developed LC-MS/MS method showed high reproducibility, as evident from the relative standard deviation (RSD) values for intra-day and inter-day variability being 1.0–6.8% and 2.0–7.7% for phenolic acids and 3.7–7.4% and 1.5–8.1% for flavonoids, respectively, and thus may be applied for simultaneous determination of phenolic acids and flavonoids in Chinese herb and nutraceuticals.

## 1. Introduction

*Taraxacum formosanum*, a Chinese medicinal herb grown in Taiwan, has been reported to exhibit several biological activities including antiproliferation of hepatoma cell [1], anti-oxidation [2] and anti-inflammation [3]. These health-promoting effects may be attributed to the presence of bioactive compounds such as flavonoids and phenolic acids in the root, leaf and flower of *T. formosanum* [4]. However, their amount and variety in *T. formosanum* remain uncertain and need to be investigated.

Phenolic acids are a group of secondary metabolites widely distributed in plants and several studies have reported their inhibition effect on the growth of pathogens and cancer cells [5–7]. For instance, a total of 15 phenolic acids detected in a Mexican plant *Quercus resinosa* were shown to be responsible for the growth inhibition of cervical cancer cell Hela [5]. Likewise, Ayaz *et al.* [6] demonstrated the effective contribution of 9 and 10 phenolic acids in the leaf and seed of kale towards scavenging DPPH free radicals and inhibiting the growth of different bacterial species such as *Staphylococcus aureus*, *Enterococcus faecalis*, *Bacillus subtilis* and *Moraxella catarrhalis*. Similar to phenolic acids, flavonoids are secondary metabolites, which are widely distributed as glycosides in fruits, vegetables, flowers and seeds for protection against damage caused by ultraviolet, insect, fungus and pathogen [8]. The bioactive role of flavonoids as an anti-cancer, anti-bacterial, anti-oxidation and anti-inflammatory agent has been well documented. Tsai *et al.* [9] demonstrated that the antiproliferative effect of *Gynostemma pentaphyllum* towards hepatoma cell HepG2 was mainly due to presence of quercetin and kaempferol [9]. Also, the isoflavones prepared from soybean cake were shown to be effective against lipopolysaccharide-induced inflammation in mice [10].

The extraction of flavonoids or phenolic acids is usually carried out by using polar solvents, such as hot water, methanol, ethanol, acetone or ethyl acetate, either alone or in combination [11,12]. For extraction of flavonoids from *Lycium Chinese* Mill fruit, Qian *et al.* [12] compared the extraction efficiency of water (100%), ethanol-water (50:50 or 95:5, v/v) and found ethanol-water (95:5, v/v) to provide the highest yield. Similarly, Hu and Kitts [13] employed ethanol-water (70:30, v/v) for extraction of flavonoids from dandelion flower (*Taraxacum officinale*) and partitioning with water and ethyl acetate yielded high amounts of luteolin and luteolin-7-glucoside, respectively. Alternatively, a mixture of methanol-water (70:30, v/v) was used to extract flavonol glycoside from onion [14]. Like flavonoids, the yield of phenolic acids from plants can be varied by using different proportion mixtures of water and ethanol. A high yield of caffeic acid derivatives was obtained from *Echinacea purpurea* by employing a 60:40 (v/v) mixture of ethanol and water as extraction solvent [15]. Yu *et al.* [16] compared four different extraction methods, namely, hot water extraction, acid hydrolysis, hydrolysis by acid and α-amylase and mixture of acid, α-amylase and cellulose, and a high amount of phenolic acids could be obtained from barley by using a combination of acid, α-amylase and cellulose. After extraction, the crude extract is frequently subjected to purification by solid-phase extraction (SPE) to fractionate flavonoids and phenolic acids. A solid-phase extraction (SPE) cartridge containing 500 mg of polyamide was used to purify flavonoids from the ethanolic extract of onion [14], while a Strata C18-E (500 mg) cartridge was employed to purify flavonoids and phenolic acids from the ethanolic extract of *Lycium barbarum* [17]. Apparently, the extraction and purification conditions can vary depending on the plant variety.

A HPLC-MS technique is often used for separation, identification and quantitation of flavonoids and phenolic acids in plants. A total of 9 phenolic acids was separated from the leaves of Chinese sweet potato (*Ipomea batatas*) within 60 min by employing a gradient mobile phase of water/acetonitrile/glacial acetic acid (980/20/5, v/v/v, pH 2.68) and acetonitrile/glacial acetic acid (1000/5, v/v) with flow rate at 3 mL/min and detection at 325 nm [18]. However, the solvent system is quite complex and resolution remains inadequate as co-elution of peaks occurred. In a recent study, Herchi *et al.* [19] developed a gradient solvent system of 0.5% acetic acid (A) and acetonitrile (B) to separate 5 phenolic acids in flax seed oil within 35 min with flow rate at 0.8 mL/min and detection by electrospray ionization (ESI)-time of flight (TOF)-mass spectrometry (MS). However, the number of phenolic acids separated is limited. For flavonoids in *G. pentaphyllum*, a total of 8 were resolved within 45 min by a Phenomenex C_18_ column (250 × 4.6 mm I.D., particle size 5 μm) and a gradient solvent system of 0.1% formic acid solution with flow rate at 1.0 mL/min, detection at 280 nm and identification by ESI-MS [20]. Likewise, a total of 16 flavonoids in *T. officinale* (dandelion), including 8 flavones and 8 flavonol glycosides, were separated within 70 min by a Phenomenex C_18_ column (150 × 3.0 mm I.D., particle size 4 μm) and a gradient mobile phase of 0.5% acetic acid in water/acetonitrile (50:50, v/v) and 2% acetic acid in water with flow rate at 0.4 mL/min and detection by ESI-MS [21]. However, the amount and variety of phenolic acids and flavonoids in a specific variety of *T. formosanum* (dandelion) in Taiwan, remains unknown and needs exploration. Thus, this study was undertaken to develop an HPLC-MS-MS method for identification and HPLC-DAD method for quantification of phenolic acids and flavonoids in *T. formosanum* species of Taiwan.

## 2. Results and Discussion

### 2.1. Comparison of Extraction Solvents

**Figure 1:**
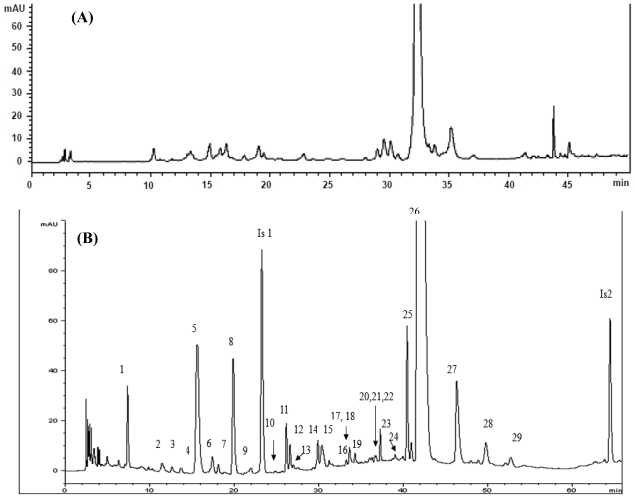
HPLC chromatogram of phenolic acids and flavonoids extracted using 100% methanol (**A**) and ethanol-water (1:1, v/v) (B) from *Taraxacum formosanum.* Column, Gemini C_18_; mobile phase, 0.1% formic acid in water and ACN; flow rate, 1 mL/min; detection wavelength, 280 nm. The peak identification is shown in Table 1.

As mentioned in the preceding section, two extractions solvent systems, methanol (100%) and ethanol-water (1:1, v/v), were compared for extraction efficiency of phenolic acids and flavonoids in *T. formosanum*. After HPLC analysis, 1:1 (v/v) ethanol-water solvent mixture was found superior to 100% methanol, as a larger number of phenolic acid and flavonoid peaks appeared in the chromatogram (Figure 1A,B). Thus, the solvent system of ethanol-water (1:1, v/v) was adopted for subsequent experiments.

### 2.2. Comparison of Various Mobile Phases in HPLC

Usually, the mobile phases employed for separation of phenolic acids and/or flavonoids include water in combination with methanol or acetonitrile in gradient mode. Additionally, a modifier such as formic acid, acetic acid, trifluoroacetic acid or phosphoric acid was added to avoid peak tailing [17,20]. Therefore, in our experiment, various mobile phase combinations containing methanol or acetonitrile with 0.1% formic acid as modifier were compared. After various trial studies, the most appropriate solvent system was composed of 0.1% formic acid in water (A) and acetonitrile (B) with the following gradient elution: 92% A and 8% B initially, maintained for 10 min, raised to 14% B in 24 min, 23% B in 35 min, 24% B in 44 min, maintained for 12 min, increased to 32% B in 60 min, 37% B in 66 min and returned to 8% B in 68 min. A total of 29 phenolic acids and flavonoids in *T. formosanum* were separated within 68 min with flow rate at 1.0 mL/min and detection at 280 nm (Figure 1B). Table 1 shows retention time (*t*_R_), retention factor (*k*), separation factor (α) and peak purity of various phenolic acids and flavonoids in *T. formosanum*. The *k* value for all the peaks ranged from 2.06 to 21.19, revealing a proper solvent strength was controlled. Likewise, the α values for all the peaks were greater than 1 (1.01–1.84), indicating that a good selectivity of mobile phase to sample components was achieved. With the exception of caffeoyl hexoside (peak 2) and luteolin hexoside hexoside (peak 17), the purities of all the other phenolic acids and flavonoids were higher than 90% (Table 1).

### 2.3. Comparison of Various Elution Volumes in SPE

**Figure 2:**
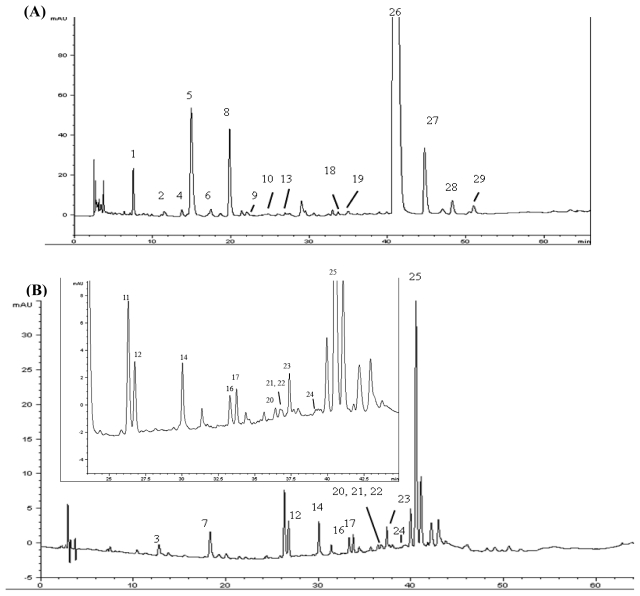
HPLC chromatogram of phenolic acids and flavonoids fractions purified using a SPE cartridge with a mobile phase of (**A**) 20 mL H_2_O and (**B**) 5 mL methanol. The peak identification is shown in Table 2. The inset chromatogram in Figure 2B shows a closer view of the peaks between retention time 25 and 45 min.

Various elution volumes involving 5–20 mL deionized water for phenolic acids and 2–5 mL methanol (100%) for flavonoids were compared for elution efficiency by using a Strata-C18-E cartridge and subjected to HPLC analysis. The most appropriate elution volume for complete elution of phenolic acids and flavonoids was 20 mL of deionized water and 5 mL of methanol, respectively. Figure 2 shows the HPLC chromatograms of phenolic acids in phenolic fraction (A) and flavonoids in flavonoid fraction (B). It was found after identification that most phenolic acids were eluted in the phenolic acid fraction with the exception of caffeoyl-d-glucose (peak 3), quinic acid derivative (peak 7) and 3,5-di-*O*-caffeoylquinic acid (peak 25).

### 2.4. Identification of Various Phenolic Acids and Flavonoids in *Taraxacum formosanum*

**Figure 3:**
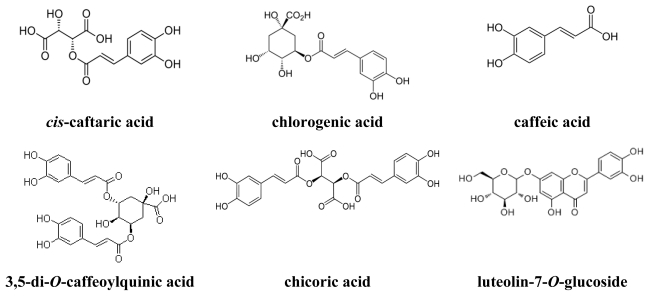
The chemical structures of phenolic acids and flavonoids positively identified by comparison with commercial standards.

Peaks 5, 8, 9, 25 and 26 were positively identified as *cis*-caftaric acid, chlorogenic acid, caffeic acid, 3,5-di-caffeoylquinic acid and chicoric acid respectively, while peak 23 as luteolin-7-*O*-glucoside by comparing the retention time, absorption and mass spectra with that of commercial standards. Figure 3 shows the chemical structures of phenolic acids and flavonoids positively identified by comparison with commercial standards. In addition, a post-column derivatization technique was used for further identification of flavonoids. The absorption characteristics of a flavonoid compound may vary depending on the type of complex it forms with the shift reagents used in the post-column derivatization method. Accordingly, the presence of flavonoid compounds was identified in our study based on the shift in absorption maximum caused by addition of sodium acetate or aluminum chloride without or with acid [20,22]. Sodium acetate, being a weak base, can complex with 7-OH in flavones or flavonols causing a bathochromic shift (red shift) of about 5–20 nm in band II. Nevertheless, an ambiguity exists in the identification of 3′- or 4′-OH of flavones or flavonols due to formation of a shoulder and irregularity in the bathochromic shift of band I [23]. On the contrary, aluminum chloride can react with 3- or 5-OH of flavones and flavonols to form acid-stable complex. Yet, an acid-liable complex is formed on reaction with two hydroxyl groups in the ortho position. Moreover, both 3,5-dihydroxyflavones and 5-deoxy-3-hydroxyflavones can result in a red shift after reaction with aluminum chloride in the presence of acid [22]. The presence of two ortho hydroxyl groups can be identified on the basis of a bathochromic shift of 30–40 nm in band I after addition of aluminum chloride plus acid, whereas the three ortho hydroxyl groups in the B ring can be detected by a red shift of only 20 nm. Similarly, a red shift of about 60, 35–55 and 50–60 nm in band I can indicate the flavonoids to be 3-hydroxy flavones, 5-hydroxy flavones and 3,5-dihydroxy flavones, respectively [22].

**Figure 4:**
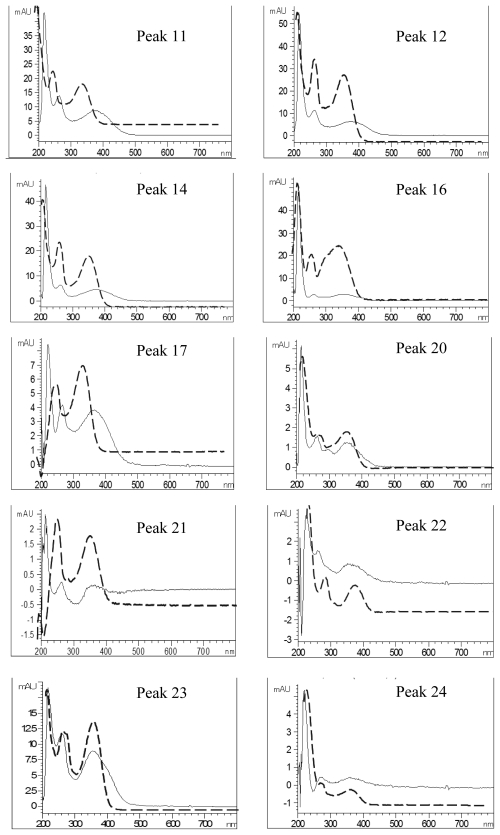
UV spectra of the flavonoid extract before (---) and after (—) post-column addition of sodium acetate reagent. Peak identification is shown in Table 1.

**Figure 5:**
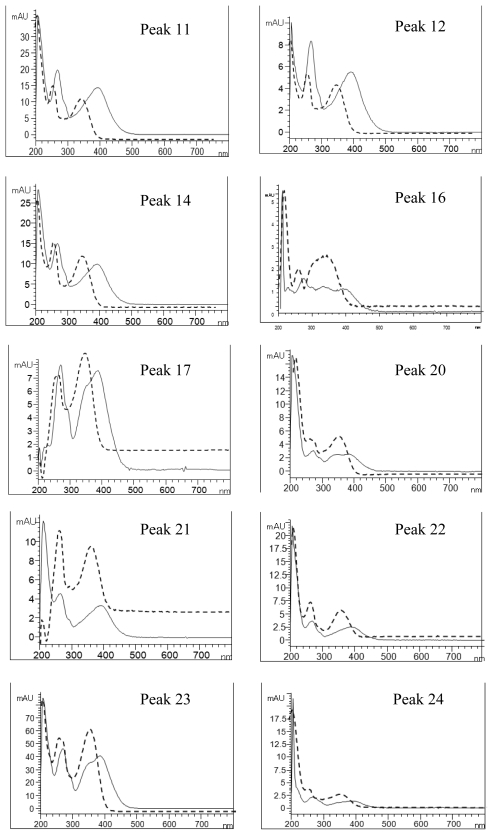
UV spectra of the flavonoid extract before (---) and after (—) post-column addition of aluminum chloride reagent without neutralization. Peak identification is shown in Table 1.

**Figure 6:**
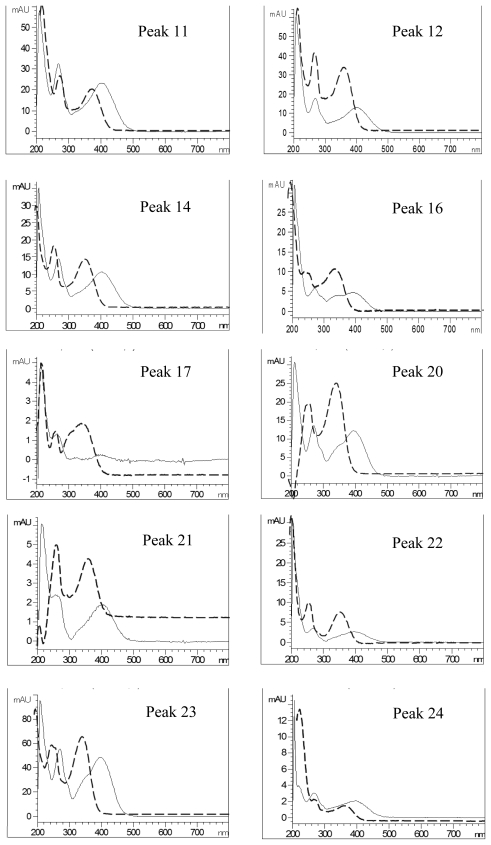
UV spectra of the flavonoid extract before (---) and after (—) post-column addition of aluminum chloride reagent with neutralization. Peak identification is shown in Table 1.

Figure 4 shows the overlaid UV spectra of each flavonoid peak before and after post-column addition of sodium acetate reagent. A bathochromic shift of 18, 20, 16, 14, 6 and 6 nm in band I was shown for peaks 11, 12, 14, 17, 23 and 24, respectively, implying the presence of hydroxy group at 3′C or 4′C, while a red shift of 14 nm (peak 24) in band II indicated the hydroxyl group at 7C position. For the other peaks, a sugar moiety may be attached to 7C. The shift in UV spectral characteristics of flavonoid peaks after post-column addition of aluminum chloride without neutralization is shown in Figure 5. The peaks 1, 12, 14, 16, 17, 20, 21, 22, 23 and 24 registered a bathochromic shift of 36, 36, 34, 38, 38, 36, 32, 34, 40 and 34 nm in band I, revealing the occurrence of hydroxyl group at C5 position. Similarly, a red shift of 44, 44, 44, 44, 52, 40, 42, 42, 50 and 44 nm observed after addition of aluminum chloride plus acid for peaks 11, 12, 14, 16, 17, 20, 21, 22, 23 and 24, respectively, revealed the existence of two hydroxyl groups at 3′C and 4′C positions (Figure 6) [24]. The afore-discussed results are all summarized in Table 2 showing the possible identities of 10 flavonoids.

For identification of phenolics and flavonoids in *T. formosanum* without commercial standards, both single quadrupole mass spectrometer with ESI mode and triple quadrupole tandem mass spectrometer (MS/MS) with multiple-reaction monitoring mode (MRM) were employed. It has been well established that the MRM mode can provide high specificity and sensitivity, and thereby the structures of unknown phenolic acids or flavonoids may be assessed based on the *m*/*z* of both precursor ion and fragment ion obtained through mass transition [25]. Moreover, the absorption spectral data were compared with that reported in the literature. Tables 3 and 4 show the mass spectral data and UV absorption maximum, respectively, for both phenolic acids and flavonoids separated from *T. formosanum*. Peak 1 showed an absorption maximum at 278 nm and an [M − H]^−^ ion at *m*/*z* 315 as well as a fragment ion at *m*/*z* 153 due to the loss of a hexose moiety, all revealing the compound to be protocatechuic acid hexoside [26,27]. Mass spectra of peak 2 displayed a parent ion at *m*/*z* 341 and two fragment ions with one at *m*/*z* 179 for caffeic acid through the loss of a hexose moiety, and the other at *m*/*z* 135 for decarboxylated caffeic acid after elimination of both hexose and CO_2_, conclusively indicating the compound to be caffeoyl hexoside. Peak 3 was identified as caffeoyl-d-glucose based on comparison of [M − H]^−^ of parent ion (*m*/*z* 339) with that reported by Shakya *et al*. [28]. Likewise, based on comparison of absorption spectra (232, 280, 310 nm) and [M − H]^−^ value (*m*/*z* 137) with that reported by Atoui *et al*. [29] and Arranz *et al*. [30], peak 4 was characterized as *p*-hydroxybenzoic acid. Peak 6 was assigned to be caffeoyl hexoside as a similar MS pattern as that of peak 2 was obtained. By comparison of absorption data with that reported by Schütz *et al*. [21], peak 7 was tentatively identified as a derivative of quinic acid and the absence of MS data may be probably caused by interference with impurities. Peak 10 was tentatively assigned to a derivative of hydroxycinnamic acid based on the [M − H]^−^ value at *m*/*z* 421 and comparison of absorption spectrum (236, 314 nm) with that reported by Sakakibara *et al*. [31]. Peak 11 was characterized to be quercetin-pentoside-hexoside as a parent ion at *m*/*z* 595 and fragment ions at *m*/*z* 433 and 301 were obtained due to a sequential loss of hexose [M − H − hexose] and pentose [M − H − hexose − pentose] moieties [21,32]. The mass pattern of peak 13 showed an [M − H]^−^ ion at *m*/*z* 441 and fragment ions at *m*/*z* 279 and 235, representing elimination of caffeic acid moiety and caffeic acid plus CO_2_ molecule, respectively, and therefore the compound was assigned to be a derivative of quinic acid [21]. The compound quercetin-pentoside-hexoside was assigned for peak 14 as the MS pattern was similar to that of peak 11. For peak 15, MS pattern depicted a parent ion at *m*/*z* 491, which upon fragmentation produced a daughter ion at *m*/*z* 329, indicating loss of a caffeic acid moiety. Additionally, a product ion at *m*/*z* 293 was mainly due to loss of dihydroxyphenyllactic acid (198 Da) plus a H_2_O molecule, as indicated by Schütz *et al*. [21] and thus the peak was tentatively characterized as caffeoyl-dihydroxyphenyllactoyltartaric acid. This identification was based on a similar compound reported to be present in commercial dandelion root and herb juices and the identification was based on the fragment ions at *m*/*z* 329, 311 (caffeoyltartaric acid), 149 (tartaric acid), 135 (decarboxylated caffeic acid) obtained in MS^2^ experiment and a product ion at *m*/*z* 293 in MS^3^ experiment after removal of a H_2_O molecule [21]. It was further reported that the exclusion of H_2_O molecule is energetically favored amid the elongated conjugated π-system in the structure [21]. However, in our study, the product ions corresponding to tartaric acid and its derivatives are missing which may be accounted for by the difference in the collision energy involved during fragmentation.

Peak 16 was characterized as quercetin-7-*O*-hexoside-3-*O*-(malonyl) hexoside based on the parent ion at *m*/*z* 711 and fragments ions at *m*/*z* 667 and 301, representing an elimination of a CO_2_ molecule and a quercetin aglycone, respectively [33]. The presence of two hexose moieties linked to a luteolin aglycone in the structure for peak 17 was identified by the parent ion at *m*/*z* 609 and fragment ions at *m*/*z* 324 and 285 [21]. An [M − H]^−^ ion at *m*/*z* 635 and λ_max_ at 216, 246, 328 nm obtained for peak 18 was tentatively identified as a derivative of caffeic acid by comparison with the literature [21]. Peak 19 with a parent ion at *m*/*z* 473 was characterized to be a derivative of chicoric acid (dicaffeoyltartaric acid), as daughter ions were generated at *m*/*z* 311 and 293 owing to the loss of caffeic acid moiety and caffeic acid plus H_2_O molecule, respectively [21]. MS profiling of peak 20 yielded a parent ion at *m*/*z* 593 and daughter ions at *m*/*z* 308 and 285 corresponding to rutinose moiety and luteolin aglycone, revealing the compound to be luteolin-7-*O*-rutinoside. Likewise, quercetin pentoside was assigned for peak 21 based on the [M − H]^−^ value at *m*/*z* 433 and the product ion at *m*/*z* 301 representing quercetin aglycone and at *m*/*z* 132 the pentose moiety. Fragmentation of the parent ion at *m*/*z* 463 for peak 22 produced two daughter ions with one at *m*/*z* 301 corresponding to quercetin aglycone, and the other at *m*/*z* 162 to glucose moiety, revealing the compound to be quercetin hexoside [34]. Similarly, quercetin pentoside was assigned for peak 24 based on the [M − H]^−^ value at *m*/*z* 433, and a product ion at *m*/*z* 299 for quercetin aglycone due to loss of pentose. Peak 27 with a parent ion at *m*/*z* 473 was characterized as a derivative of chicoric acid based on a similar MS pattern as that of peak 19. Upon fragmentation of [M − H]^−^ ion at *m*/*z* 357 for peak 28, a fragment ion at *m*/*z* 179 was produced which corresponded to caffeic acid, indicating the compound to be a derivative of caffeic acid [35,36]. Peak 29 was identified to be caffeoyl-hexose-deoxyhexoside based on the parent ion at *m*/*z* 487 and the fragment ion obtained at *m*/*z* 308 by expulsion of caffeic acid moiety and at *m*/*z* 179 due to loss of deoxyhexose plus hexose moieties [21,37].

### 2.5. Quality Control

The intra-day and inter-day variability of various phenolic acids and flavonoids in *T. formosanum* are shown in Table 5, with the RSD values ranging 1.0%–6.8% and 2.0%–7.7% for phenolic acids and 3.7–7.4% and 1.5–8.1% for flavonoids, respectively. This outcome clearly indicated that a high reproducibility can be achieved by employing the developed analytical method. The recovery of various phenolic acid and flavonoid standards shown in Table 6 revealed that a high recovery (>90%) was obtained for most standards, including *cis*-caftaric acid, chlorogenic acid, caffeic acid, 3,5-di-caffeoylquinic acid, quercetin and chicoric acid, while for luteolin-7-*O*-glucoside, the recovery was 84.9%. The recovery data was found to correlate well with the values reported by Schütz *et al*. [21], Niranjan *et al*. [38] and Inbaraj *et al*. [17].

The limit of detection (LOD) and limit of quantitation (LOQ) for *cis*-caftaric acid, chlorogenic acid, caffeic acid, 3,5-di-caffeoylquinic acid, chicoric acid, luteolin-7-*O*-glucoside and quercetin were 50.7 and 152.2, 47.9 and 143.6, 18.8 and 56.3, 88.4 and 265.1, 95.3 and 285.9, 28.3 and 85.0 and 99.6 and 298.7 ng/mL, respectively. Both LOD and LOQ in our study were substantially lower than those reported in literature [38,39]. The LOD and LOQ of chicoric acid in dried press juice of purple coneflower were shown to be 1100 and 3500 ng/mL, respectively [39]. In another study, Niranjan *et al*. [38] determined polyphenols in *Artemisia pallens* L. and the LOD and LOQ for chlorogenic acid, caffeic acid and quercetin were reported to be 1220 and 2260, 980 and 1460 and 1300 and 2400 ng/mL, respectively, Thus, it is apparent that the LC-MS method developed in our study is more sensitive than the other reported methods.

For quantitation, the following linear regression equations obtained from the calibration curve of each standard was used: y = 0.7597x − 0.2688 (*R*^2^ = 0.9968) for chicoric acid, y = 0.7385x − 0.0102 (*R*^2^ = 0.9999) for caffeic acid, y = 0.4953x − 0.1277 (*R*^2^ = 0.9968) for chlorogenic acid, y = 0.3854x − 0.176 (*R*^2^ = 0.9957) for 3,5-di-caffeoylquinic acid, y = 0.5237x − 0.0569 (*R*^2^ = 0.9959) for *cis*-caftaric acid, y = 0.3868x + 0.0105 (*R*^2^ = 0.9987) for luteolin-7-*O*-glucoside and y = 0.3356x − 0.018 (*R*^2^ = 0.9979) for quercetin. The contents of various flavonoids and phenolic acids are shown in Table 4, with the former ranging 9.9–325.8 μg/g and the latter 14.1–10870.4 μg/g (Table 4). Of the various phenolic acids and flavonoids quantified, chicoric acid and quercetin-pentoside-hexoside dominated contributing 10,870.4 and 325.8 μg/g to the total content, respectively.

## 3. Experimental Section

### 3.1. Materials

*Taraxacum formosanum* Kitam was procured from a local drug store in Taipei, Taiwan. After freeze-drying, *T. formosanum* was powdered and packed in several plastic bags. Then, they were sealed under vacuum and stored at −20 °C until further use. Phenolic acid standards, including *cis*-caftaric acid and chicoric acid, were purchased from Chromadex Co. (Santa Ana, CA, USA) and Extrasynthese Co. (Genay, France), respectively. 3,5-Di-caffeoylquinic acid was from Alexis Co. (San Diego, CA, USA), while chlorogenic acid, caffeic acid and syringic acid were from Sigma (St. Louis, MO, USA). Flavonoid standards luteolin-7-*O*-glucoside was from Extrasynthese Co., and both quercetin and (±) naringenin were from Sigma (St. Louis, MO, USA).

The HPLC-grade solvents methanol and acetonitrile were from Merck Co. (Darmstadt, Germany), while both ethanol and ethyl acetate were from Lab-Scan Co. (Gliwice, Poland). Formic acid was from Riedel-de Haën Co. (Seelze, Germany). Ethanol (95%) was from Taiwan Tobacco and Wine Bureau (Tainan, Taiwan). Deionized water was obtained by a Milli-Q water purification system from Millipore Co. (Bedford, MA, USA). The Strata C18-E cartridge (500 mg/3 mL, 55 μm, 70 Å) was from Phenomenex Co. (Torrance, CA, USA). Sodium hydroxide was from Riedel-de Haën Co. Aluminum chloride (AlCl_3_·6H_2_O) and sodium acetate were from Nacalai Tesque Co. (Kyoto, Japan). Glass filter paper GA-55 (diameter 110 mm, particle size 0.6 μm) was from Advantec Co. (Saijyo, Ehime, Japan). Polypropenyl cotton was from Applied Separation Co. (Allentown, PA, USA). Two HPLC columns, Gemini C18 (250 × 4.6 mm I.D., particle size 5 μm) was from Phenomenex Co. and Vydac 201TP54 C18 (250 × 4.6 mm I.D., particle size 5 μm) was from Vydac Co. (Hesperia, CA, USA).

### 3.2. Instrumentation

The HPLC-MS system (Agilent Technologies 1100 series, Palo Alto, CA, USA) is composed of a G1379A on-line degasser, a G1316A column temperature controller, a G1311A quaternary pump, a G1312A binary pump, a G1315B photodiode-array detector, a G1314A UV/Vis detector, and a 6130 single quadrupole MS detector with multi-mode ion source (ESI and APCI). Also, an API 3200 triple quadrupole LC-MS/MS from Applied Biosystem Co. (Carlsbad, CA, USA) was used. The Sorvall RC5C high-speed centrifuge was from Du Pont Co. (Wilmington, DL, USA), the 2210R-DTH model sonicator from Branson Co. (Danbury, CT, USA), the FD 24 freeze-dryer 24 from Gin-Min Co. (Taipei, Taiwan) and the rotary evaporator (N-1) from Eyela Co. (Tokyo, Japan).

### 3.3. Extraction and Purification

A method based on Kao *et al*. [20] was modified and used for extraction of flavonoids and phenolic acids from *T. formosanum*. A 0.25 g of *T. formosanum* powder sample was mixed with 15 mL of methanol (100%) or ethanol-water (50:50, v/v) to compare the extraction efficiency. The mixture was then shaken at 60 °C for 3 h, centrifuged at 10,000 rpm for 30 min and the supernatant was evaporated to dryness under vacuum using a rotary evaporator. The residue was dissolved in 10 mL of deionized water. Next, the crude extract was subjected to purification in a SPE cartridge based on a method described by Inbaraj *et al*. [17]. Initially, 5 mL of crude extract was poured into a vial and adjusted to pH 7 with 2% sodium hydroxide. Then 1 mL was collected and poured into a C_18_ cartridge (500 mg/3 mL, 55 μm, 70 Å), which was previously activated sequentially with 10 mL each of methanol and deionized water. The phenolic acid fraction was eluted with 15 mL of deionized water, whereas the flavonoid fraction with 5 mL of methanol (100%). The volume of eluents was optimized for complete elution by evaluating 5, 10, 15 and 20 mL of deionized water for phenolic acids and 2, 3, 4 and 5 mL of methanol (100%) for flavonoids. Each eluate was then evaporated to dryness and dissolved in 1 mL of deionized water for phenolic acid fraction and 1 mL of 50% methanol for flavonoid fraction.

### 3.4. HPLC Separation

Two HPLC columns, namely Vydac 201TP54 C18 and Phenomenex Gemini C18, were compared for separation efficiency of phenolic acids and flavonoids from *T. formosanum*. The conditions for HPLC separation was based on a previous study by Kao *et al*. [20] and are: 91% of 0.1% formic acid (A) and 9% methanol (B) initially, raised to 12% B in 3 min, 28% B in 10 min, 33% B in 15 min, 39% B in 23 min, 45% B in 27 min, 48% B in 30 min, 49% B in 35 min, 68% B in 40 min and returned to the initial solvent ratio in 45 min, with flow rate at 1 mL/min and detection wavelength at 280 nm and column temperature at 35 °C. Both *k* (retention factor) and α (separation factor) were used to assess the separation efficiency of various mobile phases. The purity of each peak was automatically determined from the Agilent G2180A Spectral Evaluation Software Data Management System.

### 3.5. Identification

Various phenolic acids and flavonoids in *T. formosanum* were identified by comparing the retention times, absorption spectra (200–600 nm) and mass spectra of unknown peaks with the reference standards. A single quadrupole mass spectrometer with ESI mode (negative mode) was used for detection with scanning range between *m*/*z* 100 and 1000, drying gas flow 6 mL/min, nebulizer pressure 60 psi, dry gas temperature 300 °C, vaporizer temperature 250 °C, capillary voltage 3500 V, charging voltage 2000 V and fragmentor voltage 200 V. In addition, a triple quadrupole LC-MS/MS with ESI mode was used for further identification with curtain gas 20 arbitrary units, collision gas 5 arbitrary units, ion spray voltage 4500 V, dry gas temperature 550 °C, ion source gas pressure 1 (60 psi), ion source gas pressure 2 (50 psi), declustering potential 25 V, entrance potential 10 V, collision energy 20 V and collision cell exit potential 5 V. In addition, a post-column derivatization technique was employed for further identification of flavonoids [24]. In brief, two HPLC pumps were connected in series after the column for pumping the derivatizing agent containing 0.3 M of aluminum chloride solution or 0.5 M of sodium acetate solution, with pH being adjusted to neutral with 0.02 M of sodium hydroxide. After HPLC separation, the eluate of each peak was mixed with the derivatizing agent and allowed to enter into the reaction coil (1 m × 0.5 mm I.D.) for reaction at 80 °C and into the photodiode-array detector for UV detection.

### 3.6. Quantitation

An internal standard (IS) syringic acid was used to quantify phenolic acids by dissolving in acetonitrile/water (1:1, v/v), whereas (±) naringenin used to quantify flavonoids by dissolving in the same solvent. Next, 5 concentrations of 7.8, 15.6, 31.3, 62.5 and 250 μg/mL of *cis*-caftaric acid or chicoric acid standard were prepared in acetonitrile/water (1:1, v/v) separately, while 7.8, 15.6, 31.3, 62.5 and 125 μg/mL of chlorogenic acid standard was prepared in the same solvent. Likewise, 5 concentrations of 0.81, 1.6, 12.5, 25 and 50 μg/mL for caffeic acid standard and 7.8, 12.5, 25, 62.5 and 250 μg/mL for 3,5-di-caffeoylquinic acid were prepared separately. Then, to each standard solution, syringic acid was added to make-up a final IS concentration of 25 μg/mL. For quantitation of flavonoids, 5 concentrations of 3.9, 7.8, 15.6, 31.3 and 62.5 μg/mL of luteolin-7-*O*-glucoside in 70% methanol and 1.3, 2.5, 5.0, 10.0 and 20.0 μg/mL of quercetin in 100% methanol were prepared separately. Then, each standard solution was mixed with (±) naringenin to obtain a final IS concentration of 20 μg/mL. Next, 20 μL of each concentration sample was injected into HPLC-DAD twice, and the standard curves were prepared by plotting concentration ratio against area ratio. Each phenolic acid and flavonoid in *T. formosanum* was quantified using a formula reported by Inbaraj *et al*. [17] as given below:

(1) $$\text{Phenolic acid or flavonoid }(\mu \text{g}/\text{g})=\frac{\left[\left(\frac{\text{As}}{\text{Ai}}\right)\times \text{a}+\text{b}\right]\times \text{Ci}\times \text{V}\times \text{DF}÷\text{R}}{\text{Ws}}$$

wherein, As: peak area of phenolic acid or flavonoid; Ai: peak area of internal standard; a: slope of calibration curve; b: intercept of calibration curve; Ci: concentration of internal standard; V: volume of extract; DF: dilution factor; R: recovery; Ws: weight of sample (g).

### 3.7. Quality Control

According to International Conference on Harmonization [40], both intra-day and inter-day variability were measured for assessing the reproducibility. The intra-day variability was determined by injecting a sample 3 times each in the morning, afternoon and evening on the same day for a total of 9 replicates, whereas the inter-day variability was estimated by injecting a sample 3 times in a day and repeated for 3 days. Both standard deviation (SD) and relative standard deviation (RSD %) were calculated for inter-day and intra-day variability results.

Accuracy of the method was validated by measuring the recovery of 1 ml of 100 μg/mL each of *cis-*caftaric acid, chlorogenic acid, caffeic acid, 3,5-di-caffeoylquinic acid, chicoric acid, quercetin and luteolin-7-*O*-glucoside spiked into 0.25 g of *T. formosanum* sample separately. After extraction, purification and HPLC analysis, the recovery of each phenolic acid or flavonoid was obtained based on the amount after HPLC (spiked amount minus original amount) divided by the amount before HPLC (spiked amount).

For determination of LOD and LOQ, 3 concentrations of *cis*-caftaric acid (80, 100 and 1000 ng/mL), chlorogenic acid (500, 2000 and 4000 ng/mL), caffeic acid (40, 500 and 1000 ng/mL), 3,5-dicaffeoylquinic acid (750, 1000 and 2000 ng/mL), chicoric acid (250, 750 and 1500 ng/mL), luteolin-7-*O*-glucoside (40, 80 and 375 ng/mL) and quercetin (650, 1000 and 2000 ng/mL) were prepared. Each concentration was injected into HPLC 3 times and the standard curves were obtained by plotting concentration against peak height. Both LOD and LOQ were determined based on the following formula [40]:

(2) $$\delta ={\text{N}}_{\text{p}-\text{p}}/5$$

(3) LOD=3.3×(δ/S)

(4) LOQ=3×LOD

wherein, N_p-p_ is the maximum noise height and S is the slope of each standard curve.

### 3.8. Statistical Analysis

All the analyses were carried out in duplicate and the data are expressed as mean ± standard deviation. The regression equations and correlation coefficient (*R*^2^) were obtained directly from the Microsoft Excel 2003 software data management system [41].

## 4. Conclusions

An HPLC-MS-MS method was developed to determine various phenolic acids and flavonoids in *T. formosanum*. A total of 29 compounds, including 19 phenolic acids and 10 flavonoids, were separated by employing a Gemini C_18_ column and a gradient mobile phase of 0.1% formic acid and acetonitrile with flow rate at 1.0 mL/min and detection at 280 nm. Identification was carried out based on the retention behavior as well as absorption and mass spectral characteristics. Internal standards syringic acid for phenolic acid and naringenin for flavonoids were used to quantification. A high recovery and reproducibility suggest the validity of this method for application to other Chinese herbs and nutraceuticals.

## Figures and Tables

**Table 1:** Retention time (*t*_R_), retention factor (*k*), separation factor (α), and peak purity of phenolic acids and flavonoids extracted from *Taraxacum formosanum.*

Peak No.	Identity	Retention Time (*t*_R_, min)	Retention Factor (*k*)	Separation Factor (α)	Peak Purity (%)
1	Protocatechuic acid hexoside	7.397	2.06	1.84 (1,2) a	99.9
2	Caffeoyl hexoside	11.596	3.79	1.84 (1,2) a	72.9
3	Caffeoyl-d-glucose	12.781	4.28	1.13 (2,3) a	99.5
4	*p*-Hydroxybenzoic acid	13.86	4.73	1.10 (3,4) a	97.2
5	*cis*-Caftaric acid b	16.11	5.66	1.20 (4,5) a	99.9
6	Caffeoyl hexoside	17.58	6.26	1.11 (5,6) a	95.5
7	Quinic acid derivative	18.253	6.54	1.04 (6,7) a	99.8
8	Chlorogenic acid b	20.098	7.30	1.12 (7,8) a	99.9
9	Caffeic acid b	22.151	8.15	1.12 (8,9) a	95.0
10	Hydroxycinnamic acid derivative	25.042	9.35	1.15 (9,10 a	98.2
11	Quercetin-pentoside-hexoside	26.287	9.86	1.06 (10,11) a	99.9
12	Quercetin-hexoside-hexoside	26.729	10.05	1.02 (11,12) a	99.9
13	Quinic acid derivative	27.165	10.23	1.02 (12,13) a	97.2
14	Quercetin-pentoside-hexoside	30.031	11.41	1.12 (13,14) a	90.2
15	Caffeoyldihydroxyphenyllactoyltartaric acid	31.097	11.85	1.04 (14,15) a	90.2
16	Quercetin-7-*O*-hexoside-3-*O*-(malonyl)hexoside	33.45	12.82	1.08 (15,16) a	85.8
17	Luteolin hexoside hexoside	33.754	12.95	1.01 (16,17) a	79.8
18	Caffeic acid derivative	34.063	13.08	1.01 (17,18) a	98.9
19	Chicoric acid derivative	34.63	13.31	1.02 (18,19) a	95.5
20	Luteolin-7-*O*-rutinoside	36.458	14.07	1.06 (19,20) a	99.2
21	Quercetin pentoside	36.745	14.18	1.01 (20,21) a	99.2
22	Quercetin hexoside	36.908	14.25	1.00 (21,22) a	99.7
23	Luteolin-7-*O*-glucoside b	37.407	14.46	1.01 (22,23) a	99.9
24	Quercetin pentoside	39.198	15.20	1.05 (23,24) a	80.9
25	3,5-Di-*O*-caffeoylquinic acid b	40.647	15.80	1.04 (24,25) a	82.8
26	Chicoric acid b	42.28	16.47	1.04 (25,26) a	99.1
27	Chicoric acid derivative	47.19	18.50	1.12 (26,27) a	99.8
28	Caffeic acid derivative	50.687	19.95	1.08 (27,28) a	93.8
29	Caffeoyl hexose-deoxyhexoside	53.709	21.19	1.06 (28,29) a	98.3

a Numbers in parentheses represent peak numbers;

b Compounds conclusively identified by comparing retention time, absorption and mass spectra with that of commercial standards.

**Table 2:** On-line UV spectral data of the *Taraxacum formosanum* flavonoids obtained in the absence and presence of shift reagents.

Peak No.	Identity	Eluent UV Spectra (nm)		NaOAc+NaOH-Shifted UV Spectra (nm)		AlCl^3^-Shifted UV Spectra (nm)		AlCl^3^+NaOH-Shifted UV Spectra (nm)	

		Band I	Band II	Band I	Band II	Band I	Band II	Band I	Band II
11	Quercetin-pentoside-hexoside	358	260	376	264	394	268	402	270
12	Quercetin-hexoside-hexoside	358	260	378	264	394	268	402	270
14	Quercetin-pentoside-hexoside	358	260	374	262	392	268	404	268
16	Quercetin-7-*O*-hexoside-3-*O*-(malonyl)hexoside	354	260	356	264	392	268	398 (328) a	266
17	Luteolin hexoside hexoside	348	260	362	264	386 (356) a	292	400 (348) a	270
20	Luteolin-7-*O*-rutinoside	350	260	354	262	386 (342) a	272	390(344) a	272
21	Quercetin pentoside	360	258	360	262	392 (286) a	266	402	260
22	Quercetin hexoside	356	262	356	262	390 (286) a	268	398	268
23	Luteolin-7-*O*-glucoside	348	260	354	260	388 (354) a	270	398 (352) a	270
24	Quercetin pentoside	356	258	362	272	390	268	400	268

a Values in parentheses represent shoulder.

**Table 3:** Mass spectral data for tentative identification of phenolic acids and flavonoids in *Taraxacum formosanum*.

Peak No.	Retention Time (min)	Identity	[M − H]^−^ (On-Line) (Parent Ion)	Fragment Ions (On-Line, MRM Mode) (Daughter Ion)	[M − H]^−^ (Reported) (Parent Ion)	Fragment Ions (Reported) (Daughter Ion)
1	7.397	Protocatechuic acid hexoside	315 b	153 [M − H − hexose]	315 b	153 b
2	11.596	Caffeoyl hexoside	341 c	179 [M − H − hexose],135 [M − H − hexose − CO_2_]	341 c	179, 135 c
3	12.781	Caffeoyl-d-glucose	339 d	-	339 d	-
4	13.86	*p*-Hydroxybenzoic acid	137 e	-	137 e	-
5	16.11	*cis*-Caftaric acid a	311 c	179 [M − H − tartaric], 149 [M − H − caffeoyl]	311 c	149, 179 c
6	17.58	Caffeoyl hexoside	341 c	179 [M − H − hexose], 135 [M − H − hexose − CO_2_]	341 c	179, 135 c
7	18.253	Quinic acid derivative	-	-	-	-
8	20.098	Chlorogenic acid a	353 c	191 [M − H − caffeoyl], 179 [M − H − quinic]	353 c	191, 179 c
9	22.151	Caffeic acid a	179 c	135 [M − H − CO_2_]	179 c	135 c
10	25.042	Hydroxycinnamic acid derivative	421	-	-	-
11	26.287	Quercetin-pentoside-hexoside	595 c	433 [M − H − hexose], 301 [M − H − hexose − pentose]	595 c	433, 301 c
12	26.729	Quercetin-hexoside-hexoside	625 c	343, 301 [M − H − 2 hexose]	625 c	343, 301 c
13	27.165	Quinic acid derivative	441 c	279 [M − H − caffeoyl], 235 [M − H − caffeoyl − CO_2_]	441 c	279, 235 c
14	30.031	Quercetin-pentoside-hexoside	595 c	433 [M − H − hexose]	595 c	433 c
15	31.097	Caffeoyl-dihydroxyphenyllactoyl-tartaric acid	491 c	329 [M − H − caffeoyl], 293 [M − H − dihydroxyphenyl lactoyltartaric acid − H_2_O]	491 c	329, 293 c
16	33.45	Quercetin-7-*O*-hexoside-3-*O*-(malonyl)hexoside	711 f	667 [M − H − CO_2_], 301 [M − H − hexose − malonyl − hexose]	711 f	667, 301 f
17	33.754	Luteolin hexoside hexoside	609 c	285 [M − H − 2 hexose]	609 c	285 c
18	34.063	Caffeic acid derivative	635 c	-	635 c	-
19	34.63	Chicoric acid derivative	473 c	311 [M − H − caffeoyl], 293 [M − H − caffeoyl − H_2_O]	473 c	311, 293 c
20	36.458	Luteolin-7-*O*-rutinoside	593 c	285 [M − H − rutinose]	593 c	285 c
21	36.745	Quercetin pentoside	433 c	301 [M − H − pentose]	433 c	301 c
22	36.908	Quercetin hexoside	463 g	301 [M − H − hexose]	463 g	301 g
23	37.407	Luteolin-7-*O*-glucoside a	447 c	285 [M − H − hexose]	447 c	285 c
24	39.198	Quercetin pentoside	433 c	-	433 c	-
25	40.647	3,5-Di-*O*-caffeoylquinic acid a	515 c	353 [M − H − caffeoyl], 173 [M − H − caffeoyl − quinic]	515 c	353, 173 c
26	42.28	Chicoric acid a	473 c	311 [M − H − caffeoyl]	473 c	-
27	47.19	Chicoric acid derivative	473 c	293 [M − H − caffeoyl − H_2_O]	473 c	-
28	50.687	Caffeic acid derivative	357 h	179	357 h	179 h
29	53.709	Caffeoyl hexose-deoxyhexoside	487 i	308 [M − H − caffeoyl], 179 [M − H − deoxyhexose − hexose]	487 i	179 i

a Compound conclusively identified by comparison of MS spectral data of unknown peaks with authentic standards;

b Based on a reference by Fang *et al*. [26];

c Based on a reference by Schütz *et al*. [21];

d Based on a reference by Shakya *et al*. [28];

e Based on a reference by Arranz *et al*. [30];

f Based on a reference by Gouveia *et al*. [33];

g Based on a reference by Mertz *et al*. [34];

h Based on a reference by Arakawa *et al*. [35];

i Based on a reference by Rivera-Pastrana *et al*. [37].

**Table 4:** UV spectral data and content of flavonoids and phenolic acids on dry weight basis in *Taraxacum formosanum.*

Peak No.	Identity	λ_max_ (On-Line)	λ_max_ (Reported)	Content (μg/g)
1	Protocatechuic acid hexoside	220, 278	257, 291 b	149.1 ± 3.41
2	Caffeoyl hexoside	226, 294, 318	234, 288sh, 297 c	49.2 ± 1.95
3	Caffeoyl-d-glucose	222, 286, 338	-	26.3 ± 0.64
4	*p*-Hydroxybenzoic acid	232, 280, 310	278, 310sh d	26.3 ± 1
5	*cis*-Caftaric acid a	218, 244, 302, 326	232, 277, 321 c	1227.3 ± 31.71
6	Caffeoyl hexoside	214, 222, 290	233, 291 c	752.4 ± 5.14
7	Quinic acid derivative	222, 264	230, 266 c	204.3 ± 7.63
8	Chlorogenic acid a	218, 240, 298sh, 324	236, 303sh, 326 c	837.2 ± 16.66
9	Caffeic acid a	248, 298, 324	241, 305sh, 323 c	39.1 ± 1.96
10	Hydroxycinnamic acid derivative	236, 314	241, 291, 319 e	14.1 ± 0.25
11	Quercetin-pentoside-hexoside	208, 260, 358	231, 260,358 c	325.8 ± 12.11
12	Quercetin-hexoside-hexoside	208, 260, 358	230,261, 358 c	176.8 ± 11.18
13	Quinic acid derivative	220, 266	230, 266 c	173.3 ± 6.19
14	Quercetin-pentoside-hexoside	212, 260, 358	231, 260, 358 c	192.7 ± 7.96
15	Caffeoyl-dihydroxyphenyllactoyl-tartaric acid	220, 288, 326	246, 300sh, 332 c	135.0 ± 2.17
16	Quercetin-7-*O*-hexoside-3-*O*-(malonyl)hexoside	206, 260, 354	-	60.0 ± 4.43
17	Luteolin hexoside hexoside	210, 260, 348	255, 266sh, 347 c	31.0 ± 1.84
18	Caffeic acid derivative	216, 246, 328	240, 310sh, 325 c	29.0 ± 1.74
19	Chicoric acid derivative	212, 292, 326	242, 305sh, 328 c	225.4 ± 2.25
20	Luteolin-7-*O*-rutinoside	206, 260, 350	255, 266sh, 348 c	26.7 ± 1.01
21	Quercetin pentoside	208, 258, 360	-	75.6 ± 3.93
22	Quercetin hexoside	210, 262, 356	256, 300sh, 354 f	12.4 ± 4.42
23	Luteolin-7-*O*-glucoside a	208, 260, 348	255, 266sh, 347 c	175.9 ± 9.44
24	Quercetin pentoside	212, 258, 356	-	9.9 ± 0.48
25	3,5-Di-*O*-caffeoylquinic acid a	220, 244, 300sh, 326	243, 303sh, 327 c	989.3 ± 22.99
26	Chicoric acid a	220, 244, 304sh, 328	242, 305sh, 328 c	10870.4 ± 150.05
27	Chicoric acid derivative	246, 302, 328	242, 305sh, 328 c	653.4 ± 7.27
28	Caffeic acid derivative	212, 230, 314	-	120.5 ± 6.09
29	Caffeoyl hexose-deoxyhexoside	220, 244, 330sh, 328	290, 320 g	51.8 ± 3.51

a Compound conclusively identified by comparison of UV spectra of unknown peaks with authentic standards;

b Based on a reference by Fang *et al*. [27];

c Based on a reference by Schütz *et al*. [21];

d Based on a reference by Atoui *et al*. [29];

e Based on a reference by Sakakibara *et al*. [31];

f Based on a reference by Mertz *et al*. [34];

g Based on a reference by Rivera-Pastrana *et al*. [37].

**Table 5:** Intra-day and inter-day variability of phenolic acids and flavonoids in *Taraxacum formosanum* as determined by HPLC-DAD.

Peak No.	Phenolic Acid/Flavonoid	Intra-Day Variability a		Inter-Day Variability a	

		Mean (μg/g) ± SD	RSD (%)	Mean (μg/g) ± SD	RSD (%)
1	Protocatechuic acid hexoside	149.1 ± 3.4	2.3	147.8 ± 9.1	6.2
2	Caffeoyl hexoside	49.2 ± 2.0	4.0	51.2 ± 2.8	5.5
3	Caffeoyl-d-glucose	26.3 ± 0.6	2.4	26.9 ± 1.1	4.3
4	*p*-Hydroxybenzoic acid	16.6 ± 1.0	6.0	15.9 ± 1.2	7.5
5	*cis*-Caftaric acid	1105.3 ± 31.7	2.9	1096.7 ± 32.0	2.9
6	Caffeoyl hexoside	84.9 ± 5.1	6.1	78.0 ± 5.8	7.5
7	Quinic acid derivative	191.5 ± 7.6	4.0	187.1 ± 3.7	2.0
8	Chlorogenic acid	784.9 ± 16.7	2.1	760.2 ± 18.6	2.5
9	Caffeic acid	39.1 ± 2.0	5.0	38.5 ± 2.6	6.8
10	Hydroxycinnamic acid derivative	14.1 ± 0.3	1.7	14.6 ± 0.8	5.7
11	Quercetin-pentoside-hexoside	325.8 ± 12.1	3.7	329.8 ± 14.7	4.4
12	Quercetin-hexoside-hexoside	176.8 ± 11.2	6.3	175.5 ± 9.9	5.7
13	Quinic acid derivative	162.4 ± 6.2	3.8	164.2 ± 7.3	4.4
14	Quercetin-pentoside-hexoside	192.7 ± 8.0	4.1	188.8 ± 6.8	3.6
15	Caffeoyldihydroxyphenyllactoyltartaric acid	135.0 ± 2.2	1.6	117.6 ± 6.9	5.8
16	Quercetin-7-*O*-hexoside-3-*O*-(malonyl)hexoside	59.9 ± 4.4	7.4	56.0 ± 3.9	7.0
17	Luteolin hexoside hexoside	26.3 ± 1.8	7.0	28.4 ± 1.6	5.6
18	Caffeic acid derivative	28.9 ± 1.7	6.0	30.6 ± 2.0	6.6
19	Chicoric acid derivative	215.4 ± 2.3	1.0	219.8 ± 4.3	2.0
20	Luteolin-7-*O*-rutinoside	11.4 ± 0.7	6.1	14.7 ± 1.1	7.1
21	Quercetin pentoside	64.1 ± 3.9	6.1	70.4 ± 5.1	7.3
22	Quercetin hexoside	60.5 ± 4.4	7.3	60.6 ± 4.9	8.1
23	Luteolin-7-*O*-glucoside	149.3 ± 9.4	6.3	139.6 ± 2.1	1.5
24	Quercetin pentoside	8.4 ± 0.5	5.7	7.9 ± 0.2	2.5
25	3,5-Di-*O*-caffeoylquinic acid	890.9 ± 23.0	2.6	857.3 ± 65.8	7.7
26	Chicoric acid	10390.0 ± 150.1	1.4	10392.4 ± 429.7	4.1
27	Chicoric acid derivative	624.5 ± 7.3	1.2	621.1 ± 25.1	4.0
28	Caffeic acid derivative	120.4 ± 6.1	5.1	110.0 ± 7.5	6.9
29	Caffeoyl hexose-deoxyhexoside	51.8 ± 3.5	6.8	52.4 ± 2.7	5.2

a Mean of duplicate analyses ± standard deviation.

**Table 6:** Recovery of phenolic acids and flavonoids as determined by HPLC-DAD.

Phenolic Acid/Flavonoid	Original (μg)	Spiked (μg)	Found (μg)	Recovery (%)	Mean ± SD (%)	RSD (%)
*cis*-Caftaric acid	28.9	16.8	43.7	88.3	90.1 ± 2.4	2.7
	27.6	16.8	43.1	91.8		
Chlorogenic acid	15.3	20.7	34.5	92.7	93.8 ± 1.5	1.6
	15.3	20.7	34.9	94.8		
Caffeic acid	3.0	20.3	22.0	93.6	94.1 ± 0.7	0.7
	2.7	20.3	21.9	93.7		
Luteolin-7-*O*-glucoside	2.0	18.6	17.9	85.0	84.9 ± 0.3	0.4
	2.0	18.6	17.8	84.7		
3,5-Di-caffeoylquinic acid	23.1	20.9	42.8	94.5	93.1 ± 1.9	2.0
	23.6	20.9	42.8	91.8		
Quercetin	0	20.1	18.8	93.6	95.3 ± 2.5	2.6
	0	20.1	19.5	97.0		
Chicoric acid	317.1	25.8	342.2	97.5	95.6 ± 2.7	2.8
	318.0	25.8	342.2	93.7		
